# Comprehensive perioperative management of a giant neurofibroma complicated by hemorrhagic shock: a case report

**DOI:** 10.3389/fsurg.2026.1794599

**Published:** 2026-07-02

**Authors:** Jiye Sang, Guojun Zhao, Yanhong Zhang, Liming Sun, Mingming Zhao

**Affiliations:** 1 Affiliated Hospital of Chengde Medical University, Operating Room, Chengde, China; 2 Affiliated Hospital of Chengde Medical University, Chengde, China

**Keywords:** hemorrhagic shock, multidisciplinary care, neurofibroma, neurofibromatosis type 1, perioperative care

## Abstract

Giant neurofibromas are rare benign peripheral nerve sheath tumors, most often occurring in association with neurofibromatosis type I. Tumor rupture may be followed by massive bleeding and hemorrhagic shock, posing life-threatening challenges during perioperative management. This report describes the multidisciplinary perioperative care of a patient with a ruptured giant neurofibroma complicated by hemorrhagic shock. A 39-year-old man with a history of congenital neurofibromatosis presented with acute swelling and pain in the right lumbar region and abdomen following trauma. Computed tomography revealed a massive retroperitoneal hematoma secondary to tumor rupture. The patient underwent emergency debridement and hemostasis under general anesthesia. Intraoperatively, approximately 3,000 mL of clotted blood was evacuated, and diffuse bleeding from the tumor cavity was controlled by suture ligation, electrocautery, and compression packing. Due to severe hemorrhagic shock, the patient required transfusion support, vasoactive drugs, mechanical ventilation, and intensive care. The surgical team focused on precise intraoperative positioning, thermal management, hemodynamic monitoring, transfusion coordination, aseptic technique, and postoperative wound compression and infection prevention. The patient's bleeding was temporarily controlled by compression and intensive supportive therapy. Subsequent multidisciplinary consultation guided further management. The patient achieved hemodynamic stabilization but required prolonged critical care due to the large wound area and ongoing risk of infection. This case highlights the pivotal role of perioperative in managing catastrophic bleeding in giant neurofibroma surgery. Individualized assessment, close coordination with surgical and anesthesia teams, and evidence-based interventions were crucial for maintaining stability and optimizing patient outcomes.

## 1 Introduction

Neurofibromas are benign peripheral nerve sheath tumors commonly associated with neurofibromatosis type I (NF1) (1). NF1 is a common autosomal dominant genetic disorder with an estimated prevalence of approximately 1 in 2,500–3,000 individuals worldwide (2). Plexiform neurofibromas occur in nearly 30%–50% of patients with NF1; however, giant plexiform neurofibromas are relatively rare and can pose substantial surgical challenges because of their infiltrative growth pattern, rich vascularity, and high risk of massive intraoperative bleeding and postoperative recurrence. Tumor rupture with hemorrhage is even more uncommon and has rarely been described in previous reports.

When the area exceeds 100 cm^2^, it is referred to as Giant neurofibromas (1). Giant neurofibromas carry a substantial risk of spontaneous rupture and hemorrhage, a clinical propensity driven by multiple interconnected pathological mechanisms. These contributory factors are as follows: (1) a typically disorganized and fragile vascular architecture characterized by thin-walled, dysplastic vessels with incomplete mural structures; (2) an underlying NF1-related vasculopathy that predisposes affected individuals to the formation of intratumoral aneurysms and arteriovenous malformations; and (3) considerable mechanical stress exerted by the tumor bulk, coupled with its locally infiltrative growth pattern, which may erode or compromise adjacent vascular structures. When rupture occurs, patients can experience sudden hemorrhage leading to hemorrhagic shock (2, 3). Such cases present extraordinary challenges for surgical teams, requiring immediate, coordinated, and precise perioperative management.

This case report aims to detail the comprehensive perioperative management strategies adopted by the multidisciplinary team in managing a 39-year-old man with a ruptured giant neurofibroma complicated by hemorrhagic shock, emphasizing the critical collaborative role of the surgical team, nursing team, anesthetic team, and critical care team in maintaining patient stability throughout different treatment phases during acute crises.

## 2 Case presentation

### 2.1 Patient information

A 39-year-old man, a freelance worker, reported striking his right flank against a hard surface. Over the next 6 days, he experienced mild right lumbar–abdominal swelling and pain. In the 4 h before presentation, the pain and swelling worsened and were accompanied by dizziness, chest tightness, and shortness of breath.

On admission, the patient appeared pale and diaphoretic, with vital signs as follows: body temperature, 36.5 °C; pulse, 94 bpm; respiratory rate, 20 breaths per min; blood pressure, 74/40 mmHg. A fixed, tender mass measuring 25 cm × 20 cm × 17 cm was palpable in the right lumbar and posterior abdominal regions. The patient had a history of congenital neurofibromatosis. Laboratory tests revealed anemia and coagulation dysfunction：Laboratory tests showed a hemoglobin level of 67.0 g/L, a hematocrit of 19.5%, a prothrombin time of 17.10 s, and a fibrinogen level of 0.512 g/L (reference range: 2–4 g/L).Abdominal computed tomography showed posterior abdominal wall swelling with an associated hematoma (Figure 1, panel 4). Based on the clinical presentation and imaging findings, a diagnosis of a ruptured neurofibroma with active bleeding and hemorrhagic shock was established.

**Figure 1:**
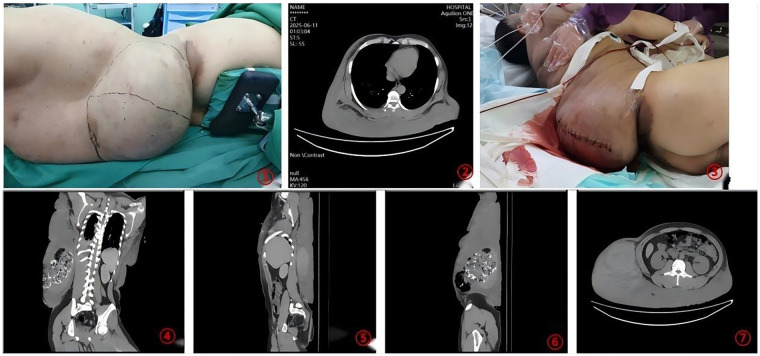
Clinical photographs and computed tomography (CT) images before and after the first emergency hemostatic procedure. (1) Preoperative gross appearance of a giant exophytic mass arising from the posterior/lateral trunk. (2) Preoperative non-contrast CT showing the lesion with a retroperitoneal hematoma consistent with tumor rupture. (3) Postoperative appearance after the first emergency operation, during which the tumor cavity was packed with gauze for compression hemostasis. (4–7) Postoperative coronal, sagittal, and axial images show the tumor mass. First postoperative CT images demonstrating the anatomic extent and configuration of the tumor mass after hemorrhage control.

### 2.2 Treatment and outcome

The patient underwent four surgical procedures during hospitalization (Figure 5).

#### 2.2.1 First surgery

An emergency operation was performed due to tumor rupture and hemorrhage (Figure 1). A 15-cm midline longitudinal incision was made over the mass and extended through all layers of the tumor capsule. Tumor rupture was identified; approximately 3,000 mL of clotted blood and dark bloody fluid were evacuated. The resulting cavity was markedly enlarged and contained multiple actively bleeding vessels along with extensive capillary oozing. Complete hemostasis was difficult to achieve.Ruptured intracavitary vessels were ligated with figure-of-eight sutures and monopolar electrocautery.

Owing to the patient's critical condition, complete hemostasis was deemed technically unfeasible and carried an unacceptably high risk. The family was informed of the situation and gave written informed consent for hemostatic packing with plans to perform definitive resection once the patient's condition improved. A tumor wall biopsy was obtained for histopathological analysis. After focal hemostasis, 6 g of hemostatic powder was applied to the cavity, which was then firmly packed with 19 dry surgical pads. The incision was closed in layers.

#### 2.2.2 Preoperative evaluation

The therapeutic priorities during this phase were the resuscitation of hemorrhagic shock, correction of concomitant coagulopathy, and maintenance of overall homeostasis, aiming to optimize the patient's condition for subsequent tumor resection surgery.A multidisciplinary consultation was organized involving Burns Department, Vascular Surgery Department, Anesthesiology, ICU, and Blood Transfusion Department.

Contrast-enhanced computed tomography angiography (CTA) was performed to evaluate the tumor's vascular supply, including major feeding arteries and suspected aberrant vessels, to inform hemostatic and surgical planning (Figure 2). The dorsal tumor was supplied by branches of the bilateral subscapular arteries, select intercostal arteries, and the distal segments of the bilateral lumbar arteries at the L2-L4 vertebral levels.

**Figure 2:**
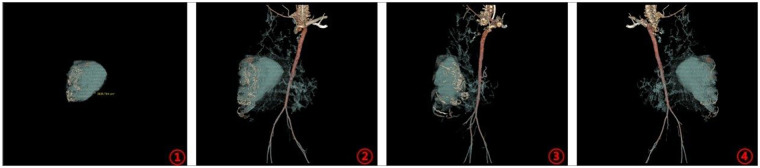
Preoperative computed tomography angiography (CTA) evaluation of tumor burden and vascular supply. (1) CTA showing the tumor volume. (2–4) CTA images showing the altered vascular anatomy and major tumor-feeding vessels.

#### 2.2.3 Second surgery

Twenty-four hours before definitive tumor resection, digital subtraction angiography (DSA) and superselective arterial embolization were performed by the endovascular team under general anesthesia.

The vascular territory and collateral circulation of the feeding arteries were identified during DSA. Superselective embolization of the tumor's main and feeding arteries was achieved using a combination of embolic materials, including coils for the main trunks and branches, as well as gelatin sponge and polyvinyl alcohol particles for small vessels.

A repeat DSA confirmed the complete occlusion of the tumor-feeding arteries, followed by compression hemostasis at the puncture site (Figure 3).

**Figure 3:**
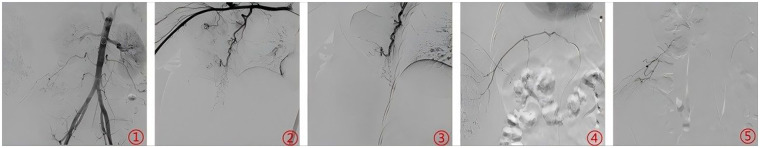
Intraoperative digital subtraction angiography and superselective arterial embolization of tumor-feeding vessels. (1–4) Serial angiograms show the arterial network with a progressive reduction in distal arterial branches. (5) Completion angiogram demonstrating the occlusion of tumor-feeding vessels.

#### 2.2.4 Third surgery

The patient was maintained in a prone position with careful padding and stabilization to protect the posterior trunk mass and facilitate surgical exposure (Figure 4, panels 1–2). A preoperative resection area measuring approximately 55 cm × 68 cm was delineated. The previous incision was reopened, and 19 gauze pads and two Vaseline gauze strips were removed from the tumor cavity. The mass consisted of yellowish-white soft tissue with mild active bleeding, which was controlled by electrocautery. To minimize intraoperative blood loss, a circumferential ligature using No. 7 silk sutures was placed along the planned incision line. Dissection began at the left margin, extending through the skin and subcutaneous tissue to the tumor surface. The left portion of the mass was elevated and progressively separated toward the spinal midline and right side, with perforating vessels individually ligated for hemostasis. The right incision revealed tissue characteristics similar to those of the left. The tumor was dissected along the muscular plane, involving the platysma, teres major, teres minor, and latissimus dorsi. At the paraspinal base, nodular plexiform neurofibromatous tissue was identified, partially invading the right latissimus dorsi. Complete excision of the affected tissue was achieved, and the specimen was set aside to harvest a split-thickness skin graft from the overlying skin. The tumor mass was mobilized along anatomical tissue planes to the level of the muscular fascia. Adequate exposure was obtained, and tumor dissection and excision were performed with active hemostatic control under direct visualization (Figure 4, panels 3–4). The excised specimen was documented, and its size was recorded (Figure 4, panel 5).

**Figure 4:**
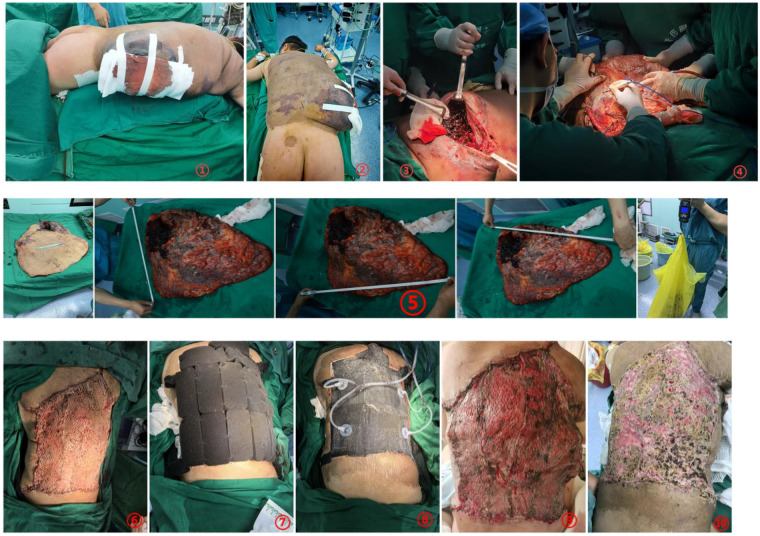
Tumor resection and wound reconstruction. (1, 2) Intraoperative prone positioning using a customized pad. (3, 4) Intraoperative views of tumor dissection and excision with hemostatic control. (5) Gross appearance and size of the excised tumor specimen. (6–8) Placement of a split-thickness meshed skin graft followed by vacuum sealing drainage (VSD)/negative pressure wound therapy. (9) Wound appearance after VSD removal on postoperative day 6.

**Figure 5:**
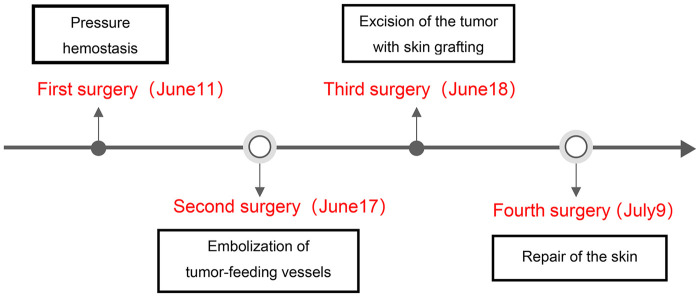
The patient underwent four surgical procedures during hospitalization.

A split-thickness skin graft (∼11% of total body surface area) was harvested from normal skin overlying the resected mass using an electric dermatome. After thorough saline irrigation, the graft was manually meshed and applied to the dorsal wound. The edges were secured with skin staples. After the wound bed was irrigated with saline and hemostasis was confirmed, a vacuum sealing drainage (VSD) system was applied over the graft for negative pressure wound therapy (NPWT), sealed with a transparent adhesive drape, and connected to a suction unit. The surgical field was then covered with sterile dressings and reinforced with an abdominal binder for compression (Figure 4, panel 6).

Given the extensive tumor size and complex dissection, the procedure lasted approximately 10 h, with an estimated blood loss of 2,000 mL. Intraoperatively, the patient received 10 units of red blood cells, 1,050 mL of plasma, and 10 units of cryoprecipitate, without any transfusion-related reactions. Hemodynamics remained stable throughout the procedure, and anesthesia was satisfactory. The patient was transferred to the intensive care unit (ICU) postoperatively in stable condition. The resected specimen weighed approximately 10.7 kg (Figure 4, panel 5).

#### 2.2.5 Fourth surgery

A fourth operation was performed to achieve debridement and re-grafting for partial graft necrosis. Split-thickness skin grafts were harvested from the thigh and applied to the wound area (Figure 4, panel 10). Most of the grafted skin pieces remained well-fixed and viable, with adherent dry crust on the surface. The residual wound area was approximately 3%, with a granulation tissue base and no purulent discharge. Preoperative Assessment: The patient was currently in stable condition, with anemia, hypoproteinemia, and electrolyte imbalances having been corrected. The residual wounds were unlikely to heal spontaneously or would require an excessively long time to heal, carried a high risk of significant post-healing scar hyperplasia, or were susceptible to severe infection. Postage stamp skin grafting was therefore indicated for wound closure.After multidisciplinary treatment and nursing care, the patient recovered well and was discharged after 51 days of hospitalization.

## 3 Perioperative management

### 3.1 Preoperative assessment and preparation

In response to the patient's unstable hemodynamics and suspected bleeding, the initial priorities were to maintain airway patency, monitor oxygen saturation, and establish rapid venous access. Central venous catheterization was performed under ultrasound guidance to enable volume resuscitation and vasoactive drug administration. Coordinated efforts by the clinical team​ ensured the expedited processing of laboratory tests, blood cross-matching, and preparation of blood products. Throughout this process, psychological support was provided to the patient, family communication was maintained, and informed consent for emergency surgery was secured.

### 3.2 Intraoperative management

#### 3.2.1 Hemostasis

As described in a case report of hemorrhage control in NF-1 (4), hemostasis prioritized the exposure of the surgical field, prompt identification of bleeding vessels, ligation, and electrocautery, followed by gauze packing and layered compression/tamponade as needed. Operative drainage was coordinated with anesthesiologists to maintain hemodynamic stability during ongoing blood loss.

#### 3.2.2 Hemodynamic and thermal management

Blood pressure, urine output, and body temperature were measured as follows. Blood pressure was continuously monitored via a radial arterial catheter. Urine output was measured hourly through an indwelling urethral catheter connected to a urine collection bag with an integrated measuring chamber. Core temperature was continuously recorded using a nasopharyngeal or esophageal temperature probe. Active warming measures included forced-air warming blankets and prewarmed irrigation solutions to prevent hypothermia-induced coagulopathy (5, 6). Intraoperative fluid resuscitation, vasoactive support, and blood component replacement were coordinated with anesthesiologists based on real-time hemodynamic parameters.

#### 3.2.3 Patient positioning and skin protection

The large tumor mass required special padding and continuous monitoring to prevent pressure injuries. Nurses placed soft silicone cushions under pressure points and adjusted limb angles to maintain adequate circulation.

#### 3.2.4 Transfusion management

The circulating nurse closely collaborated with the anesthesia team to document blood loss, prepare additional blood products, and maintain accurate records, including product type, identification number, and administration times. Timely communication with the blood bank ensured an uninterrupted supply of blood products.

### 3.3 Postoperative management

Postoperative management focused on maintaining hemodynamic stability and optimizing conditions for healing of the extensive dorsal wound. Prone positioning with scheduled axial turning was implemented to protect the surgical site, while the vacuum sealing drainage (VSD) system was carefully maintained to ensure effective wound drainage and stability. Continuous negative pressure of −50 mmHg was applied to the posterior trunk wound bed as a bolster dressing for the skin graft (7, 8), and increased to −100 to −150 mmHg when drainage exceeded 100 mL. On postoperative day 6, the dressing was removed for wound assessment, revealing satisfactory granulation tissue formation over most of the wound bed, although granulation in the upper right shoulder region was relatively poor. This area was subsequently treated with povidone-iodine disinfection and povidone-iodine-impregnated gauze dressing.

### 3.4 Patient outcomes

Surgery was performed by vascular and plastic surgeons as well as neurosurgeons. The patient recovered well without any major complications. Local recurrence and potential malignant transformation were evaluated during follow-up. The case was managed using a multidisciplinary, pre-planned operative strategy, including predefined intraoperative contingency protocols. The patient had a long-standing lumbar back mass and presented for emergency care after tumor rupture with hemorrhage. After complete resection and recovery, the patient and family reported satisfaction with the outcome.

## 4 Discussion

This case highlights the substantial perioperative challenges associated with giant neurofibromas complicated by catastrophic hemorrhagic shock, a rare but potentially fatal emergency that has been infrequently reported in recent literature. Recent studies have emphasized the role of endovascular embolization as a lifesaving intervention for spontaneous hemorrhage in plexiform neurofibromas (5, 6, 8, 9). However, compared with previously reported cases, the present case involved a massive truncal neurofibroma accompanied by profound hemodynamic instability, thereby necessitating a more complex, staged, and multidisciplinary management strategy.

The therapeutic pathway adopted in this case—consisting of emergent surgical exploration and packing, followed by delayed embolization and staged reconstruction—reflects the clinical reality that immediate definitive resection may not always be feasible in patients with severe hemodynamic compromise. Recent literature increasingly supports staged management strategies for hypervascular tumors, particularly in the presence of uncontrolled bleeding, coagulopathy, or extensive soft tissue involvement (10, 11). Although this approach reduced the immediate risk of intraoperative mortality, it also resulted in prolonged intensive care unit (ICU) admission, repeated exposure to anesthesia, and partial graft necrosis secondary to suboptimal wound bed conditions prior to embolization. These outcomes underscore the inherent trade-offs associated with damage-control strategies in complex oncologic emergencies.

Compared with earlier case reports describing single-stage resection or isolated embolization procedures, our experience suggests that successful management of giant ruptured neurofibromas depends not only on effective hemorrhage control but also on the coordinated integration of surgical, anesthetic, interventional radiology, and reconstructive expertise (9, 12). This multidisciplinary framework was particularly important for balancing aggressive hemostatic intervention with the preservation of tissue viability and reconstructive potential.

Several perioperative principles applied in this case are consistent with current evidence regarding the management of major hemorrhagic surgery. Massive transfusion protocols and active warming measures are essential for interrupting the lethal triad of hypothermia, acidosis, and coagulopathy during high-volume blood loss (13, 14). In addition, negative pressure wound therapy (NPWT) has increasingly been recognized as a valuable adjunct in complex wound management, particularly during staged reconstruction following tumor resection or in contaminated wounds (15–17). Notably, this case also highlights the importance of continuous interprofessional communication throughout all perioperative stages. Emerging evidence suggests that structured multidisciplinary collaboration improves outcomes in high-risk surgical emergencies by facilitating timely decision-making, coordinated transfusion management, and rapid adaptation of operative strategies (18, 19). In the present case, close cooperation among surgeons, anesthesiologists, intensivists, interventional radiologists, and nursing staff was critical for maintaining hemodynamic stability and ensuring continuity of care during staged treatment.

Because reports of ruptured giant neurofibromas remain scarce, optimal management strategies have not yet been standardized. Nevertheless, this case provides additional evidence supporting a flexible, patient-tailored, multidisciplinary staged approach for giant neurofibromas complicated by life-threatening hemorrhage. Further accumulation of similar cases may help establish clearer recommendations regarding the timing of embolization, definitive resection, and reconstruction in these rare but highly challenging surgical emergencies.

## 5 Conclusion

Comprehensive perioperative management was essential for the successful stabilization of this patient with a ruptured giant neurofibroma and hemorrhagic shock. Individualized nursing assessments, multidisciplinary collaboration, and protocol-based interventions were critical in controlling bleeding, preventing complications, and improving recovery. Although such events are rare, this case may inform perioperative planning and multidisciplinary coordination in high-risk presentations.

## Data Availability

The datasets presented in this article are not readily available because this is a case report. Requests to access the datasets should be directed to Jiye Sang,1057067874@qq.com.
